# Protein Deiminase 4 and CR3 Regulate *Aspergillus fumigatus* and β-Glucan-Induced Neutrophil Extracellular Trap Formation, but Hyphal Killing Is Dependent Only on CR3

**DOI:** 10.3389/fimmu.2018.01182

**Published:** 2018-05-29

**Authors:** Heather L. Clark, Serena Abbondante, Martin S. Minns, Elyse N. Greenberg, Yan Sun, Eric Pearlman

**Affiliations:** ^1^Department of Ophthalmology and Visual Sciences, Case Western Reserve University, Cleveland, OH, United States; ^2^Department of Ophthalmology, University of California Irvine, Irvine, CA, United States; ^3^Department of Physiology and Biophysics, University of California Irvine, Irvine, CA, United States

**Keywords:** *Aspergillus*, neutrophil extracellular trap, protein deiminase 4, CR3, keratitis

## Abstract

Neutrophil extracellular trap (NET) formation requires chromatin decondensation before nuclear swelling and eventual extracellular release of DNA, which occurs together with nuclear and cytoplasmic antimicrobial proteins. A key mediator of chromatin decondensation is protein deiminase 4 (PAD4), which catalyzes histone citrullination. In the current study, we examined the role of PAD4 and NETosis following activation of neutrophils by *A. fumigatus* hyphal extract or cell wall β-glucan (curdlan) and found that both induced NET release by human and murine neutrophils. Also, using blocking antibodies to CR3 and Dectin-1 together with CR3-deficient CD18^−/−^ and Dectin-1^−/−^ murine neutrophils, we found that the β-glucan receptor CR3, but not Dectin-1, was required for NET formation. NETosis was also dependent on NADPH oxidase production of reactive oxygen species (ROS). Using an antibody to citrullinated histone 3 (H3Cit) as an indicator of PAD4 activity, we show that β-glucan stimulated NETosis occurs in neutrophils from C57BL/6, but not PAD4^−/−^ mice. Similarly, a small molecule PAD4 inhibitor (GSK484) blocked NET formation by human neutrophils. Despite these observations, the ability of PAD4^−/−^ neutrophils to release calprotectin and kill *A. fumigatus* hyphae was not significantly different from C57BL/6 neutrophils, whereas CD18^−/−^ neutrophils exhibited an impaired ability to perform both functions. We also detected H3Cit in *A. fumigatus* infected C57BL/6, but not PAD4^−/−^ corneas; however, we found no difference between C57BL/6 and PAD4^−/−^ mice in either corneal disease or hyphal killing. Taken together, these findings lead us to conclude that although PAD4 together with CR3-mediated ROS production is required for NET formation in response to *A. fumigatus*, PAD4-dependent NETosis is not required for *A. fumigatus* killing either *in vitro* or during infection.

## Introduction

Neutrophil extracellular trap (NET) formation is a coordinated form of neutrophil cell death, first described by Zychlinsky and colleagues in which genomic DNA is released from neutrophils and traps and mediates killing of pathogenic bacteria, fungi, and parasites (1–3). The molecular events in this process involve protein kinase C, Raf, Mek, and Erk activation, which leads to activation of NADPH oxidase, production of reactive oxygen species (ROS), and mobilization of neutrophil elastase (NE) into the nucleus, where it functions to degrade histones, resulting in chromatin decondensation (1). NETosis also requires activation of protein deiminase 4 (PAD4) in the nucleus, which converts positively charged arginine residues to neutral citrulline. Histone citrullination also mediates chromatin decondensation and nuclear swelling (4–8).

We reported that neutrophils play a critical role in fungal killing and in corneal infections with the pathogenic molds *Aspergillus* and *Fusarium*, which are major causes of blindness and visual impairment worldwide (9, 10). We demonstrated an essential role for neutrophil NADPH oxidase and for the antimicrobial peptide calprotectin (CP) in fungal killing *in vitro* and in murine models of fungal keratitis (11, 12).

In the current study, we examined the role of PAD4 in NET formation in response to *Aspergillus fumigatus* or cell wall β-glucan and show that although PAD4 is required for NET formation *in vitro* and during corneal infection, it is not required for hyphal killing *in vitro* or during infection. Instead, we demonstrate that CR3 (CD11b/CD18), which also binds β-glucan, is required for production of reactive oxygen, hyphal killing, and CP release. We conclude that although NETs are generated by *A. fumigatus* hyphae, they are not required for hyphal killing *in vitro* or during corneal infection.

## Results

### *A. fumigatus* and β-Glucan Induce Neutrophil Extracellular Traps From Human and Mouse Neutrophils

**Figure 1:**
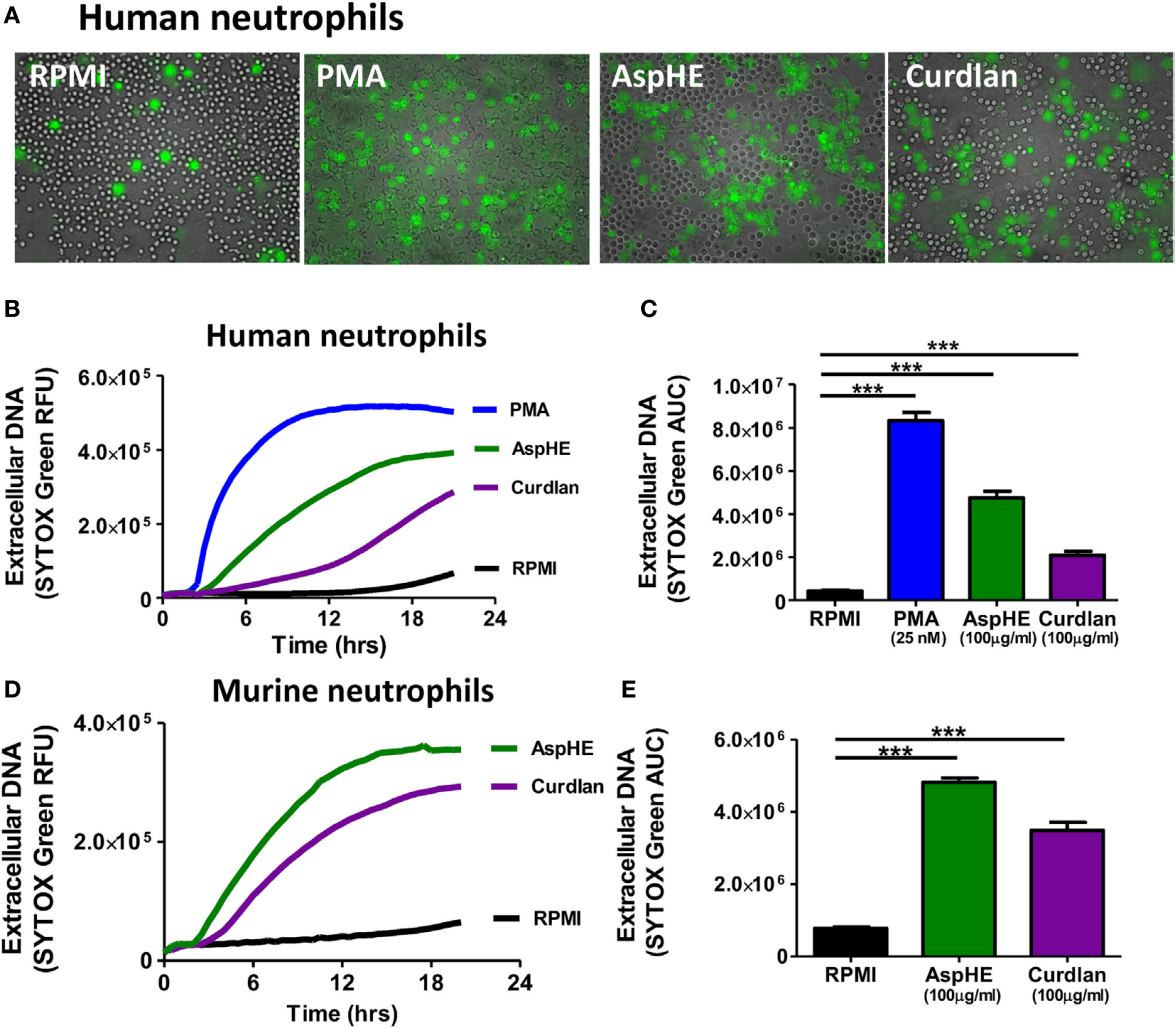
*Aspergillus fumigatus* and β-glucan induce neutrophil extracellular trap formation in human and murine neutrophils. **(A)** Representative images showing DNA release by SYTOX Green staining in human peripheral blood neutrophils 20 h after stimulation with 25 nM phorbol myristic acid (PMA), 100 μg/ml *A. fumigatus* hyphal extract (AspHE), or 100 μg/ml particulate β-glucan (curdlan); original magnification is 200×. **(B)** DNA release overtime shown as relative fluorescent units (RFU) measured by SYTOX Green; **(C)** total extracellular DNA after 20 h calculated as area under the curve (AUC). **(D,E)** Extracellular DNA from mouse peritoneal neutrophils incubated with AspHE or curdlan and quantified by SYTOX Green RFU and AUC. Asterisks (***) indicate *p* < 0.001 based on one way ANOVA with Tukey post hoc analysis. Experiments were repeated five times with similar results.

Although NETs have a distinctive appearance in fixed cells, NETs in live cell cultures are more diffuse (7), which we also found after incubating human peripheral blood neutrophils with *A. fumigatus* hyphal extract (AspHE), particulate β-glucan (curdlan), or phorbol myristic acid (PMA) when incubated with the cell-impermeable DNA stain SYTOX Green (Figure 1A).

Quantification of SYTOX Green showed that extracellular DNA increased over time (Figure 1B). Total DNA released over 20 h (area under the curve of Figure 1B) showed that all three stimuli induced a significant increase in DNA release from human peripheral blood neutrophils compared with media alone (Figure 1C). Similarly, mouse bone marrow neutrophils released DNA in response to AspHE and curdlan (Figures 1D,E), although PMA did not stimulate murine bone marrow neutrophils (not shown).

### β-Glucan-Induced NETosis Requires Production of ROS

**Figure 2:**
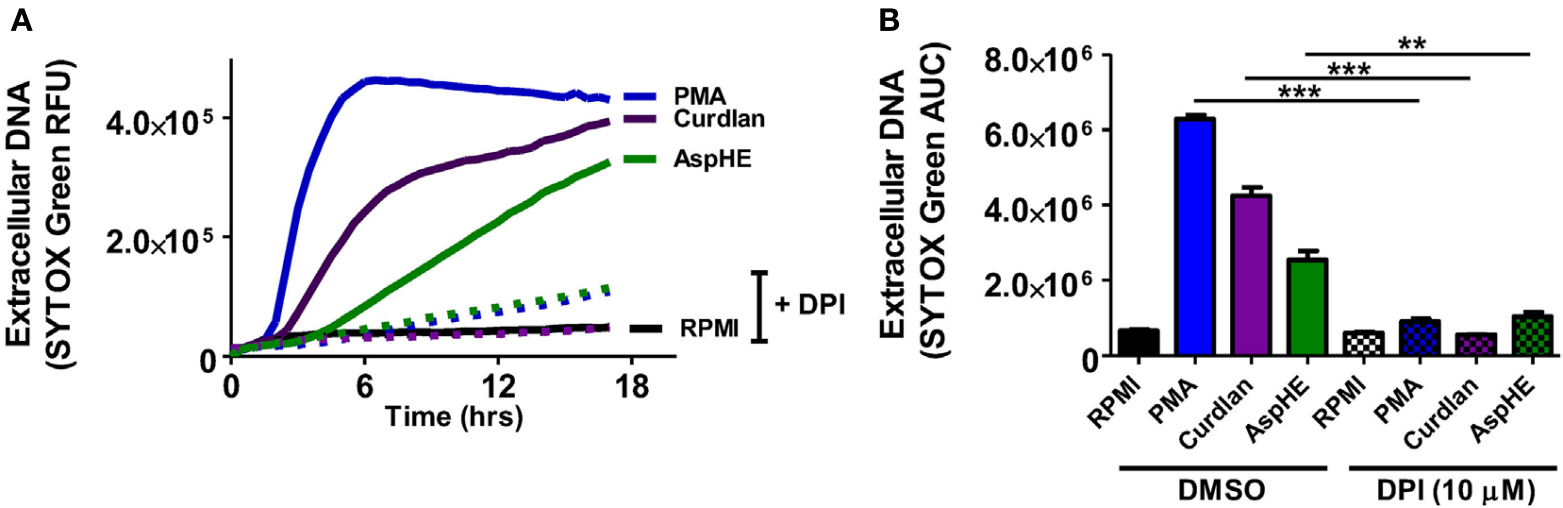
Neutrophil extracellular trap (NET) formation is dependent on reactive oxygen species (ROS). Human neutrophils were stimulated 20 h with phorbol myristic acid (PMA), AspHE, or curdlan in the presence of the ROS inhibitor diphenyl iodonium (DPI) (10 μM). NET formation was quantified by SYTOX Green over time **(A)**, and total SYTOX over 18 h is shown as area under the curve (AUC) **(B)**. ANOVA with Tukey post hoc analysis showed ***p < 0.001 and **p < 0.01. Experiments were repeated four times with similar results.

Reactive oxygen species is required for NETosis in human neutrophils following stimulation with PMA, although ROS-independent NET formation has also been reported (13–15). To examine if ROS has a role in NET formation in response to *A. fumigatus*, human neutrophils were incubated with PMA, curdlan, or AspHE in the presence of the NADPH oxidase inhibitor diphenyl iodonium (DPI). We found that DPI inhibited DNA release in response to all stimuli (Figures 2A,B), indicating a requirement for NADPH oxidase-generated ROS in NET formation in response to fungal cell wall components.

### CR3 Mediates NET Formation and ROS Production in Response to β-Glucan

C-type lectin receptors, including Dectin-1, Dectin-2, and Dectin-3 (also called macrophage c-type lectin) on murine macrophages recognize fungal cell wall components, including β-glucan and α-mannose (16). Neutrophils constitutively express the C-type lectin Dectin-1 and the β2-integrin CR3 (Mac-1, CD11b/CD18), which in addition to the complement binding I-domain, has a lectin binding domain that recognizes β-glucan (17). The lectin binding domain mediates ROS production in response to *Candida* and *Aspergillus* (11, 13), although maximal production of ROS and NETs depends on activation of both domains of this unique receptor in the presence of complement (15).

**Figure 3:**
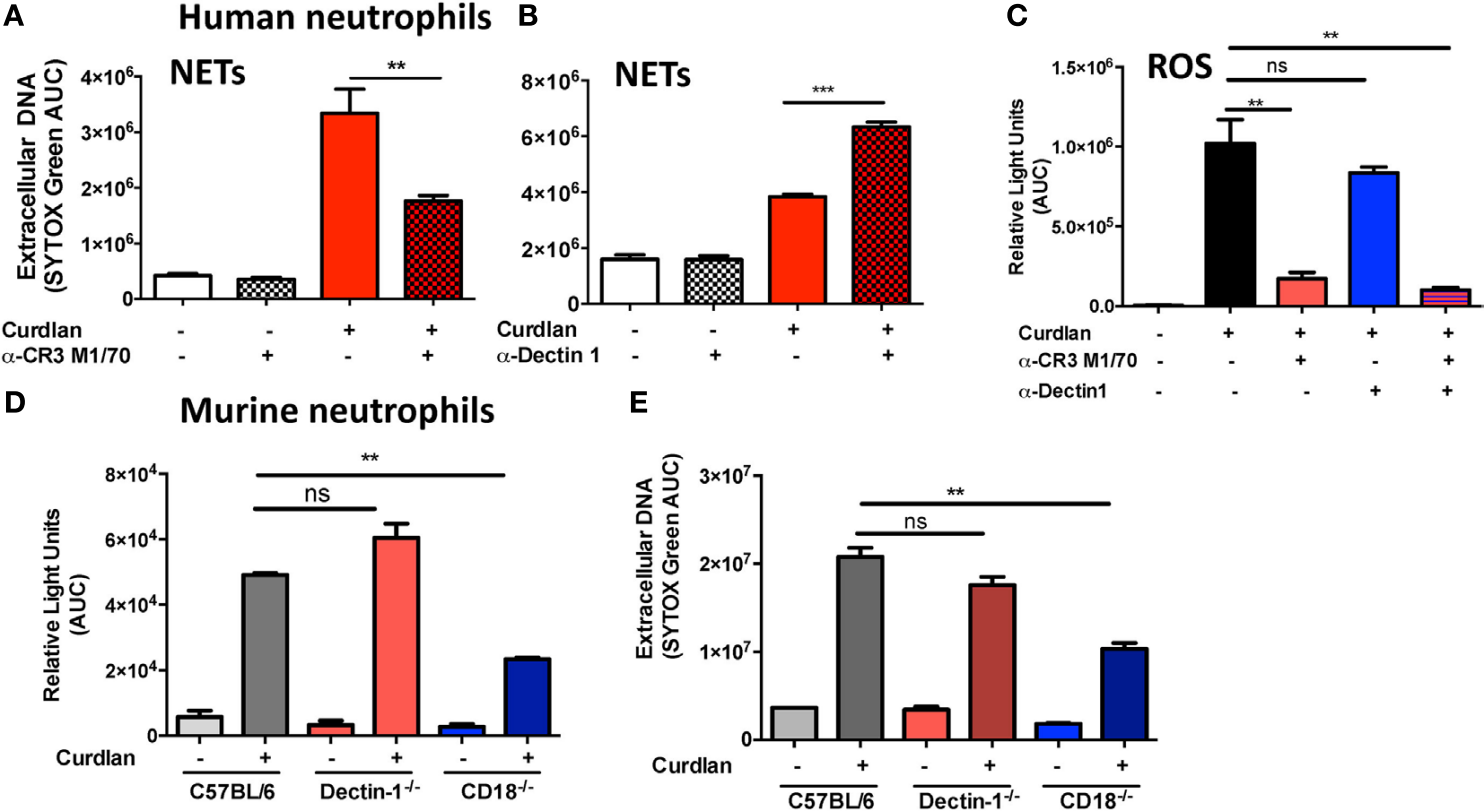
Distinct roles for CR3 and Dectin-1 in β-glucan-induced reactive oxygen species (ROS) production and neutrophil extracellular trap (NET) formation. **(A–C)** Human neutrophils; **(D,E)** murine neutrophils. Neutrophils were incubated with β-glucan (curdlan) in the presence of the anti-CR3 blocking antibody M1/70 (30 μg/ml) **(A)** or with the anti-Dectin-1 blocking antibody 22H8 (Invivogen, 20 μg/ml) **(B)**. **(A,B)** DNA release was quantified by SYTOX Green and expressed as area under the curve. **(C)** ROS production measured by luminol. **(D,E)** DNA release and ROS production by mouse bone marrow neutrophils from C57BL/6, Dectin-1^−/−^, and CD18^−/−^ mice stimulated with curdlan. *p* Values are biological replicates of at least three repeat experiments and were calculated using ANOVA with Tukey post hoc analysis (***p < 0.001, **p < 0.01, and *p < 0.05).

To determine the relative contribution of CR3 and Dectin-1 in ROS production and NET formation, human peripheral blood neutrophils were incubated with particulate β-glucan (curdlan) together with antibodies to either Dectin-1 or CR3. We found significantly reduced extracellular DNA in the presence of anti-CR3 (Figure 3A), whereas anti-Dectin-1 resulted in increased DNA release (Figure 3B), which is consistent with an earlier report on NETosis induced by *Candida* hyphae compared with yeast (18). As ROS is required for NET formation, we also examined the role of CR3 and Dectin-1 in ROS production, and found that curdlan-induced ROS production was inhibited by anti-CR3, but not anti-Dectin-1 (Figure 3C).

These findings were reproduced in bone marrow neutrophils from Dectin-1^−/−^ and from CD18^−/−^ mice, where NETosis was dependent on CD18 expression, and there was no effect of Dectin-1 deletion on β-glucan-induced NET formation or ROS production (Figures 3D,E). We therefore conclude that CR3 rather than Dectin-1 is the predominant β-glucan receptor required for ROS production and NET release in human and murine neutrophils.

### PAD4 Is Required for NET Formation Induced by β-Glucan

Conversion of arginines to neutral citrullines is mediated by the protein deimidase family of enzymes (19); however, only PAD4 is present in the nucleus, where it mediates citrullination of histones and subsequent chromatin decondensation.

**Figure 4:**
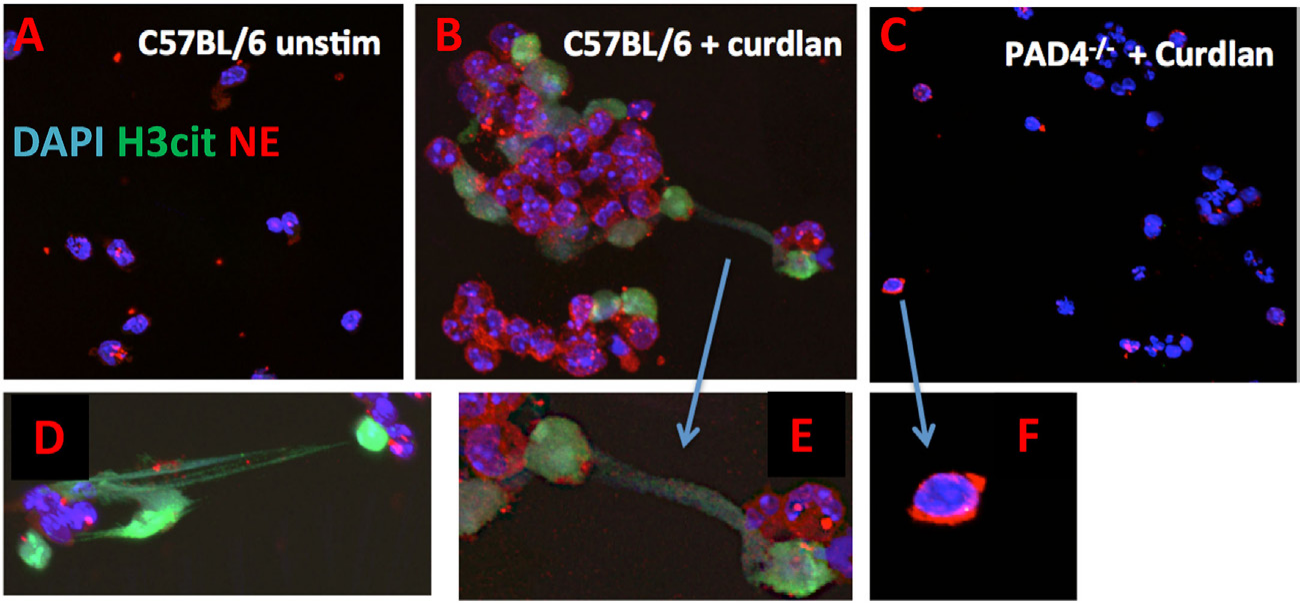
β-Glucan-induced neutrophil extracellular trap formation. Curdlan-stimulated bone marrow neutrophils from C57BL/6 and PAD4^−/−^ mice. Neutrophils were immunostained with antibodies to citrullinated histone 3 (H3Cit), neutrophil elastase (NE), and DAPI nuclear stain **(A–C)** 200×; **(D–F)** 400×.

To examine if PAD4 has a role in NET formation in response to fungal cell wall components, we visualized NET formation in β-glucan stimulated neutrophils from C57BL/6 and PAD4^−/−^ mice using antibodies to citrullinated histone H3 (H3Cit). We found that in response to curdlan, neutrophils from C57BL/6 mice generated characteristic NET structures that were also H3Cit^+^ (Figures 4A–E). In contrast, stimulated PAD4^−/−^ neutrophils retain their lobular nuclei and were negative for H3Cit (Figures 4C,F). An early stage of NETosis is NE translocation to the nucleus (4), which is clearly detected in C57BL/6 neutrophils; however, elastase remained in the cytoplasm of PAD4^−/−^ neutrophils (Figures 4C,F).

**Figure 5:**
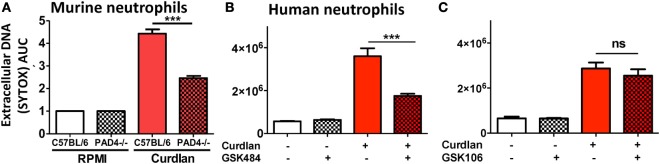
Protein deiminase 4 (PAD4)-dependent neutrophil extracellular trap formation. **(A)** Bone marrow neutrophils from C57BL/6 and PAD4^−/−^ mice were incubated 20 h with particulate β-glucan (curdlan) in RPMI, and DNA release was quantified by SYTOX Green. Values were normalized to unstimulated neutrophils (RPMI). **(B,C)** Human peripheral blood neutrophils incubated with curdlan and 10 μM specific PAD4 inhibitor GSK484 **(B)** or with the related, non-inhibitory compound GSK106 **(C)**. *p* Values are biological replicates of at least three repeat experiments and were calculated using ANOVA with Tukey post hoc analysis (***p < 0.01; **p < 0.05; ns, not significant). Experiments were repeated twice with similar results.

Protein deiminase 4-dependent NET formation by murine and human neutrophils was quantified by SYTOX green. Consistent with representative images, PAD4^−/−^ neutrophils released significantly less DNA than C57BL/6 neutrophils following incubation with curdlan (Figure 5A). To investigate if there is a role for PAD4 in NET formation by human neutrophils, we used small molecule PAD4 inhibitors that inhibit NET formation *in vitro* (19). Curdlan-stimulated human neutrophils were incubated with the PAD4 inhibitor GSK484 or with the negative control compound GSK106. As shown in Figures 5B,C, DNA release in response to *Aspergillus* was significantly reduced in the presence of GSK484, whereas the negative control GSK106 did not inhibit DNA release.

Together, these findings clearly demonstrate that PAD4 citrullination is required for NET formation by murine and human neutrophils.

### CR3 but Not PAD4 Is Required for Calprotectin Release *In Vitro*

The antimicrobial peptide calprotectin (CP; S100A8/A9) is a major component of NETs in response to *Candida albicans* and is required for NET-mediated killing (3). CP was found to be an essential NET component in controlling *A. fumigatus* following gene therapy for chronic granulomatous disease (20), and we reported that CP inhibits *A. fumigatus* hyphal growth by sequestering zinc *in vitro* and in *Aspergillus* keratitis (12).

To determine if CP is associated with NETs, we examined CP release in CR3 (CD18^−/−^) and PAD4^−/−^ neutrophils following stimulation with curdlan. Bone marrow neutrophils from C57BL/6, CD18^−/−^, and PAD4^−/−^ mice were incubated with curdlan 2h (prior to NET formation) or for 16 (after NET formation), and the CP subunit S100A8 was measured in cell-free supernatants by ELISA.

**Figure 6:**
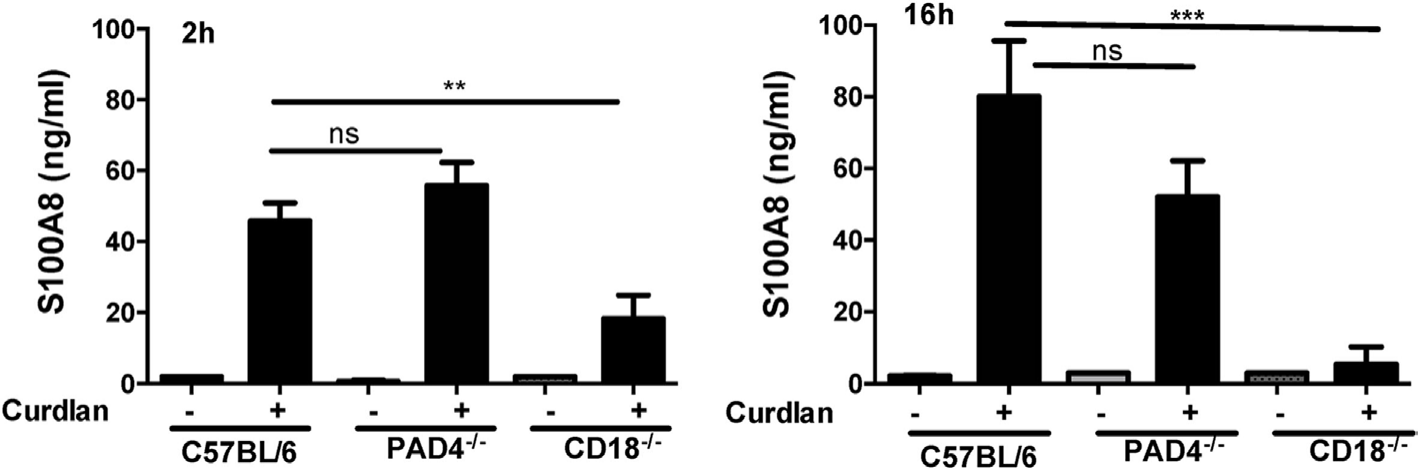
CD18-dependent calprotectin (CP) (S100A8/A9) production. Bone marrow neutrophils from C57BL/6, PAD4^−/−^, and CD18^−/−^ mice were incubated 2 or 16 h with curdlan, and CP/S100A8 was quantified by ELISA. *p* Values are biological replicates of three repeat experiments and were calculated using ANOVA using a Tukey post hoc analysis (***p < 0.001; **p < 0.01; ns, not significant). S100A8 production to AspHE was similar in all groups (Figure S1 in Supplementary Material).

Whereas there was no significant difference in S100A8 between C57BL/6 and PAD4^−/−^ neutrophils released in response to curdlan at either time point, CD18^−/−^ neutrophils produced significantly less S100A8 at both time points (Figure 6). There was no difference in production of S100A8 in response to total hyphal extracts (Figure S1 in Supplementary Material).

These data indicate that CP is released from neutrophils in the absence of PAD4-dependent NET formation, and that release in response to curdlan requires CR3 activation.

### CR3, but Not PAD4, Is Required for Neutrophil Inhibition of *A. fumigatus* Hyphal Growth

To determine whether NETs regulate fungal growth *in vitro*, 2 × 10^5^ human neutrophils were incubated with *A. fumigatus* hyphae in the presence of the anti-CR3 blocking antibody M1/70. Also, *A. fumigatus* hyphae were incubated with neutrophils from C57BL/6 and PAD4^−/−^ mice. *A. fumigatus* hyphal growth over 18 h was quantified using calcofluor white, which binds cell wall chitin, and percent fungal mass was quantified based on hyphal growth in media alone (100%).

**Figure 7:**
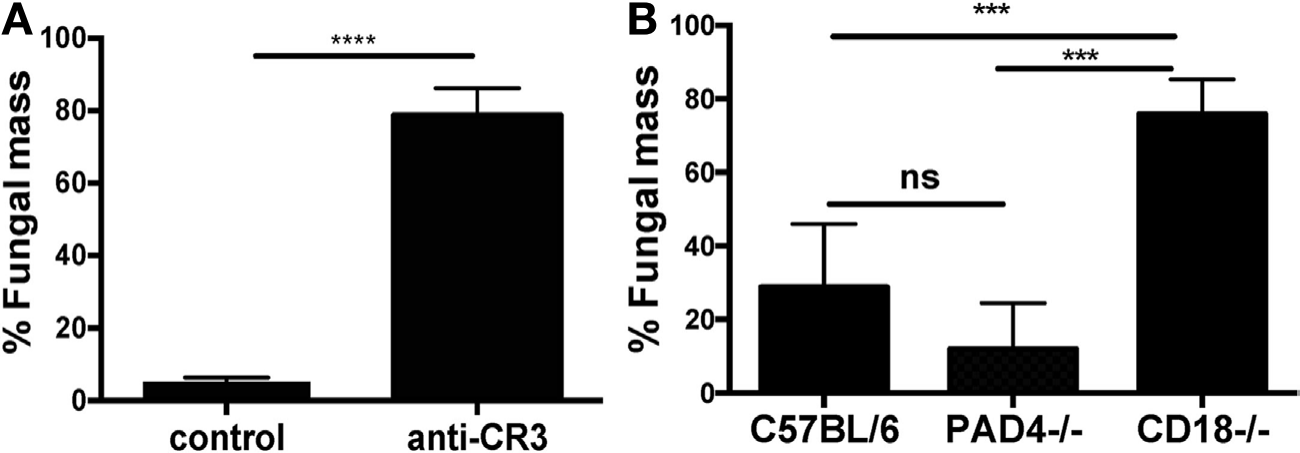
CR3, but not protein deiminase 4 (PAD4), is required for neutrophil inhibition of *Aspergillus fumigatus* hyphal growth. **(A,B)** *A. fumigatus* hyphae incubated 18 h with human neutrophils in RPMI alone or with anti-CR3 M1/70 (20 μg/ml) **(A)**. Fungal mass was quantified by calcofluor white fluorescent chitin stain and presented as percent of total hyphae in the absence of neutrophils. **(B)** Percent fungal mass in bone marrow neutrophils from C57BL/6, CD18^−/−^, or PAD4^−/−^ mice following 18 h incubation with *A. fumigatus* hyphae. *p* Values are biological replicates of three repeat experiments and were calculated using Student’s *t*-test **(A)** or ANOVA with a Tukey post hoc analysis **(B)** (****p < 0.0001; ***p < 0.001; ns, not significant).

Human neutrophils significantly inhibited fungal growth compared with hyphae grown in media alone; however, this was reversed in the presence of blocking antibody to CR3 (Figure 7A). Similarly, hyphal growth was impaired when incubated with 2 × 10^5^ C57BL/6 or PAD4^−/−^ neutrophils, but not with neutrophils from CD18^−/−^ mice (Figure 7B).

Together, these data demonstrate that CR3 signaling is required for fungal growth inhibition and/or killing by neutrophils *in vitro*, whereas there is no apparent role for PAD4.

### PAD4 Is Not Required for *A. fumigatus* Killing, Corneal Disease, or Neutrophil Recruitment to Infected Corneas

Fungal keratitis is characterized by an early and profound infiltration of neutrophils to the corneal stroma in patients (21), and in murine models where these cells are required for hyphal killing and which mediate corneal opacification (9, 10).

**Figure 8:**
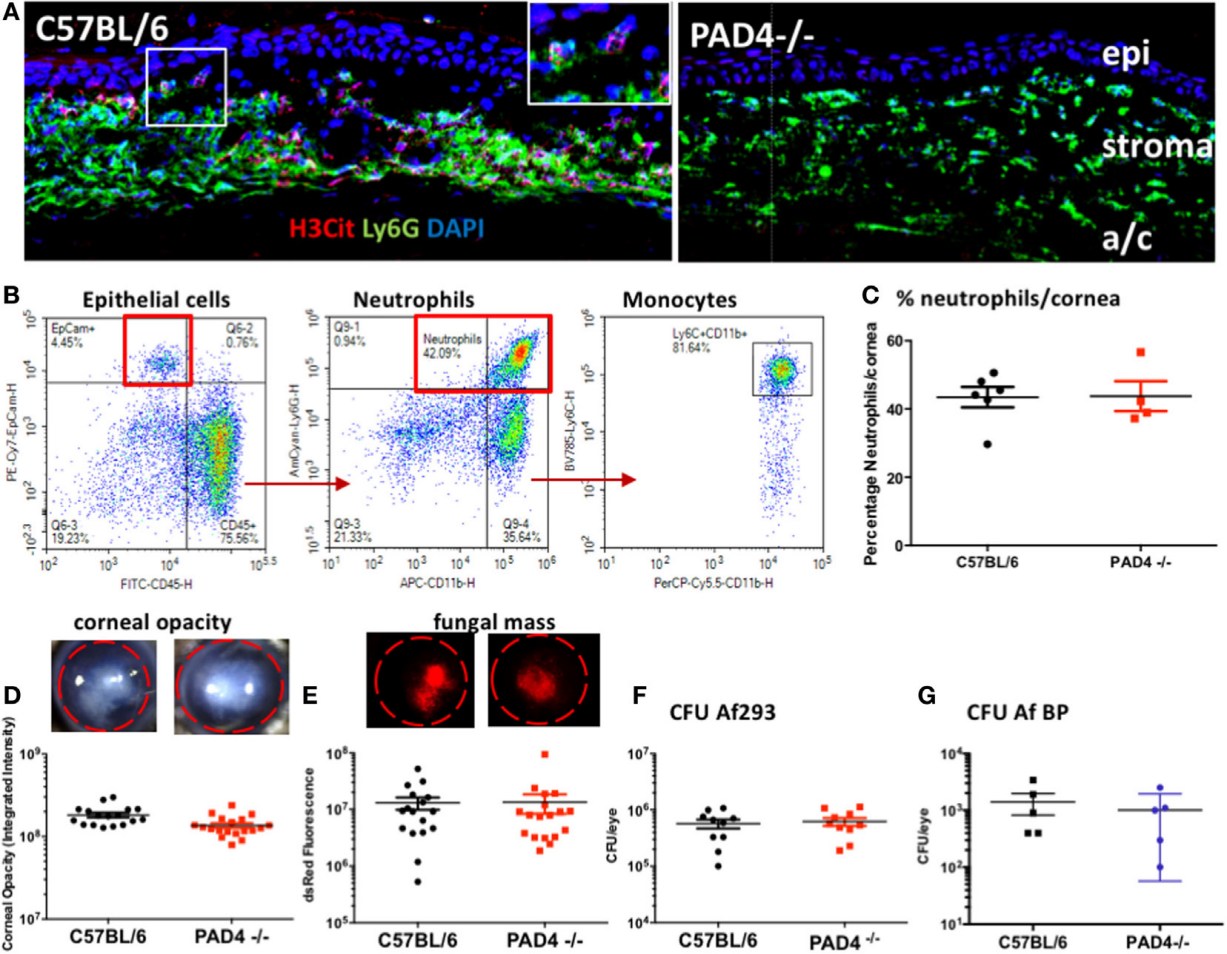
*Aspergillus fumigatus* corneal infection in PAD4^−/−^ mice. Corneas of C57BL/6 and PAD4^−/−^ mice were infected with dsRed expressing *A. fumigatus*. **(A)** Corneal sections immunostained with antibodies to Ly6G and citrullinated histone 3 (H3Cit) and counterstained with DAPI nuclear stain. Epi, stroma = corneal epithelium and corneal stroma. Highlighted area shows H3Cit staining in neutrophils. Original magnification is 400×. **(B)** Representative flow cytometry scatter plots showing total cells from infected C57BL/6 corneas that include CD45^−^ epithelial cells, CD45^+^Ly6G^+^ neutrophils, and Ly6C high monocytes. **(C)** Percent neutrophils in infected corneas from C57BL/6 and PAD4^−/−^ mice [percent Ly6G^+^ neutrophils in infected corneas (representative scatter plots from C57BL/6 and PAD4^−/−^ mice are shown in Figure S2 in Supplementary Material)]. **(D,E)** Representative corneas showing opacification and RFP expressing *A. fumigatus* hyphae. **(F,G)** Quantification of corneal opacity, dsRed as a measure of fungal mass, and CFU. Each data point represents an individual cornea. *p* > 0.05 using Student’s *t*-test analyses for panels **(C–G)**, indicating that there are no statistically significant differences between C57BL/6 and PAD4^−/−^ mice for any of these parameters. These data are representative of five repeat experiments.

To determine if PAD4 is required for NET formation in the cornea, and if NETs have a role in fungal killing at this site, corneas of C57BL/6 and PAD4^−/−^ mice were infected with 1 × 10^5^ RFP expressing *A. fumigatus* conidia (Af293), and after 48 h, corneal sections were examined for neutrophil infiltration and NET formation (H3Cit^+^). H3Cit staining was evident in corneas of *A. fumigatus* infected C57BL/6, but not PAD4^−/−^ mice (Figure 8A). Also, quantification of multiple sections showed a significant difference in H3Cit, but no significant difference in total Ly6G^+^ cells between C57BL/6 and PAD4^−/−^ mice (Figure S2 in Supplementary Material).

Total cells in infected corneas were examined by flow cytometry following collagenase digestion. As shown in Figure 8B, epithelial cells were identified as CD45^−^, EpCam^+^, which comprised <5% total cells, whereas >75% of total cells were CD45^+^. Neutrophils were identified in the CD45^+^ population as Ly6G/CD11b^+^, with the remaining CD45^+^ cells being Ly6C/CD11b^+^ monocytes. Neutrophils comprised 40–60% total cells in infected corneas; however, there was no difference in the percentage of neutrophils between infected C57BL/6 and PAD4^−/−^ corneas (Figure 8C; Figure S2 in Supplementary Material).

There were also no significant differences between infected C57BL/6 and PAD4^−/−^ mice in the severity of corneal opacification, RFP-Af293 hyphal mass or CFU (Figures 8D,E). We also found no significant difference in CFU between C57BL/6 and PAD4^−/−^ mice following infection with an *A. fumigatus* keratitis clinical isolate (Af BP) (Figures 8F,G). Collectively, these findings show that although PAD4-dependent NET formation is detected in infected corneas, they do not have an essential role in regulating fungal corneal infections.

## Discussion

A proposed sequence of events for NET formation by Zychlinsky and colleagues envisions an initial activation of neutrophils, assembly of the NADPH oxidase complex and production of ROS, resulting in bacterial entrapment and killing (1, 22). Human and mouse neutrophils with mutations or deletions in NADPH oxidase proteins cannot undergo NETosis *in vitro* or in a murine model of pulmonary aspergillosis (14). Elastase translocation to the nucleus is also required, and NETs are not detected following inhibition of elastase activity or in elastase deficient neutrophils (4).

Neutrophil extracellular traps have also been detected *in vivo* using two-photon microscopy, including in a murine model of pulmonary aspergillosis that shows hyphae in close association with neutrophils and extracellular DNA (23). These and other elegant studies leave little doubt that NETs are generated in response to bacteria, fungi and parasites. The controversy comes in to play in determining the role of NETs in killing pathogenic fungi. In the same pulmonary aspergillosis study, NETs did not kill *A. fumigatus*, although the authors suggest that NETs may have a role in inhibiting hyphal growth (23).

Human neutrophils from healthy, but not patients with chronic granulomatous disease (CGD) form NETs in response to *A. fumigatus* hyphae; however, DNAse treatment had no effect on hyphal killing in these patients, arguing against a role for NETs (24). Conversely, DNAse treatment inhibited the ability of human neutrophils to kill *A. fumigatus* germ tubes and hyphae (25, 26), thereby implicating a protective role for NETs in *A. fumigatus* infection.

In agreement with our current observations, the CGD study also showed that *A. fumigatus* conidia are recognized by CR3 rather than Dectin-1 on human neutrophils and mediates non-oxidative killing (24). This observation is supported by a report showing that Dectin-1 negatively regulates hyphae-induced NET formation by human neutrophils (18), and by an earlier study showing that human neutrophils use CR3 to form NETs in response to *C. albicans* (27). The latter study also reported that in murine neutrophils, Dectin-1 rather than CR3 mediates NETosis induced by *C. albicans*. However, we used neutrophils from Dectin-1^−/−^ and CD18^−/−^ mice to show that it is CR3 rather than Dectin-1 that mediates ROS production and NETosis, which is consistent with our earlier finding that CR3 rather than Dectin-1 that mediates ROS production by murine neutrophils (11).

Although this discrepancy may be a function of *Candida* vs. *Aspergillus*, Reichner and colleagues provide evidence of a second NETosis pathway in the presence of extracellular matrix components such as fibronectin (13, 15). Whereas the classical pathway of NETosis described by Zychlinsky requires chromatin decondensation mediated by elastase translocation to the nucleus that is ROS dependent and occurs over a period of hours (1, 22), Reichner describes a rapid process (<30 min) of NET formation induced by *Candida* hyphae, which is independent of ROS formation (15).

As our studies show elastase in the nucleus, that ROS is required for NET formation, and that this process occurs over a number of hours, we conclude that NETosis progresses through the classical pathway. However, it is possible that in the presence of extracellular matrix, the NETosis pathway regulated by matrix proteins may also be activated; indeed, this may in part explain why we found no role for PAD4 in infected corneas even while detecting PAD4-dependent H3Cit. Activation of both pathways could also explain discrepancies in the role of PAD4 described in other microbial infections (28–30).

In contrast to other NET-related mediators such as elastase and ROS, PAD4 specifically regulates histone citrullination and is unique among the protein deiminase family in being localized to the nucleus (31, 32). Therefore, the only phenotype of PAD4^−/−^ neutrophils should be an inability to form NETs so we focused on the role of PAD4 in neutrophil responses to *A. fumigatus*. Furthermore, although PAD4-dependent NETosis has been reported in mouse models of bacterial and parasitic infections, and in autoimmunity (4, 33), there are no reports on the role of PAD4 in fungal infections.

In this study, we visualized citrullinated histone H3 associated with NETs following β-glucan stimulation of C57BL/6, but not PAD4^−/−^ neutrophils, and NETosis measured by SYTOX was also dependent on PAD4. Given that β-glucan is a major cell wall surface component of molds and yeast following germination, these findings indicate that most pathogenic fungi would stimulate NET formation. However, PAD4^−/−^ neutrophils did not exhibit impaired CP release or have any role in hyphal killing *in vitro*; furthermore, although H3Cit was detected in *A. fumigatus* infected corneas, we found no difference in hyphal growth or corneal opacification between C57BL/6 and PAD4^−/−^ mice. Therefore, at least under these experimental conditions, PAD4-dependent NET formation is not required to control hyphal growth or fungal infection.

Following corneal infection with *A. fumigatus*, we found only localized H3Cit staining compared with the large neutrophil infiltrate, so it is possible that the absence of a phenotype is a consequence of relatively few NETs being formed; however, it is more likely that even though antimicrobial peptides are associated with NETs, NETosis is not required for CP release, iron sequestration or ROS production, all of which are important in controlling hyphal growth *in vitro* and in infection models (11, 12, 34). These findings are consistent with other studies in which PAD4-dependent NETs were not required to control bacterial CFU in a cecal ligation model of peritonitis (30) or for controlling influenza (29), although PAD4^−/−^ mice showed an impaired ability to control *Shigella flexneri* infection (4). Both ROS and PAD4 independent NETosis have been described for *Leishmania amazonensis* promastigotes (33).

Instead of PAD4, we found that CR3 was required for ROS production in response to curdlan and *A. fumigatus*, release of CP, and *A. fumigatus* hyphal killing. Although CR3 was also required for NET formation, these findings do not implicate NETs in fungal killing as production of ROS and CP may be independent of NETosis. Indeed, PAD4^−/−^ neutrophils released comparable levels of CP as C57BL/6 neutrophils within 2 h, which is earlier than we can detect NET formation, and indicates that CP release is NETosis independent.

Taken together, CR3 appears to be the β-glucan receptor that leads to PAD4 activation and NETosis on human and murine neutrophils, which is dependent on ROS; however, although NETs contain antimicrobial peptides, we conclude that NET formation is not required for CP release or to regulate *A. fumigatus* hyphal growth *in vitro* or in the fungal keratitis model. Furthermore, because CD18 (LFA1) binding to ICAM-1 on capillary endothelial cells is required for neutrophil attachment and extravasation, CD18^−/−^ neutrophils are not recruited to infected tissues; therefore, we were unable to examine CR3 recognition of *Aspergillus* in infected corneas.

Although the role of NETs in microbial killing appears to be controversial, NETs have also been implicated in tissue damage during infection and in autoimmune disease including rheumatoid arthritis, lupus, vasculitis, acute respiratory distress syndrome, and sepsis. This is due in part to release of histones that are abundant in NETs and which have direct cytotoxic activity (35). One example is intravenous injection of methicillin resistant *Staphylococcus aureus*, which resulted in PAD4-dependent NET formation in the liver and resulting hepatic injury (36). By contrast, we found that corneal opacity as a measure of tissue damage in fungal keratitis was not reduced in *A. fumigatus* infected PAD4^−/−^ mice, indicating that NETs do not appear to regulate blinding corneal disease related to fungal infection. NETs have been reported in *Pseudomonas aeruginosa* keratitis, although ExoS-expressing strains are more susceptible than ExoU to NET-mediated bacterial killing (37).

A recent review of NETs proposes a clear distinction between ROS and elastase-dependent NETs, which have antimicrobial activity, and a second ROS independent, PAD4-mediated mechanism of DNA release termed leukotoxic hypercitrullination, which is triggered by elevated intracellular calcium (which activates PAD4) and is associated with autoimmunity (38). Our findings reveal β-glucan-induced NET formation that is both ROS and PAD4 dependent and is associated with intranuclear elastase, thereby suggesting this dichotomy is not so clear. The same review also notes that the main purpose of PAD4-mediated histone citrullination is to alter the chromatin and regulate gene transcription, whereas elastase digestion of chromatin is also required for NETosis. Our observations that release of extracellular DNA can be blocked by inhibiting PAD4 or ROS production makes it unlikely that we are seeing two distinct pathways.

In conclusion, this study demonstrates an essential role for CR3, ROS, and PAD4 in NET formation in response to *Aspergillus* and to β-glucan but shows that PAD4-dependent NETosis is not required for either hyphal killing or for tissue damage in the cornea.

## Materials and Methods

### Ethics Statement

Human peripheral blood was collected from healthy donors between ages of 18 and 65 years in accordance with the Declaration of Helsinki guidelines and with the approval of the Institutional Review Board of the University of California (Irvine, CA, USA). Informed consent was obtained in writing from each volunteer.

All animals were treated in accordance with the guidelines provided in the Association for Research in Vision and Ophthalmology ARVO statement for the Use of Animals in Ophthalmic and Vision Research. All animal studies were conducted following approval of the Institutional Animal Care and Use Committee of Case Western Reserve University and the University of California, Irvine.

### Human Peripheral Blood Neutrophils

Whole blood RBCs were separated in 3% dextran (Sigma-Aldrich, St. Louis, MO, USA) PBS, and neutrophils were purified from remaining cells by overlay on a Ficoll (GE Healthcare) density gradient and centrifugation at 500 × *g* for 25 min. Remaining RBCs were lysed, and neutrophils were resuspended in RPMI 1640 medium. Purity (~90%) was assessed by flow cytometry using antihuman D16 and CD66b Abs (eBioscience, San Diego, CA, USA).

### Source of Mice

CD18^−/−^ mice were originally provided by Claire Doerschuk (University of North Carolina, Chapel Hill, NC, USA). Age- and sex-matched C57BL/6 mice were purchased from Jackson Laboratories (Bar Harbor, ME, USA). PAD4^−/−^ mice were originally provided by Dr. Kerri Mowen at Scripps Research Institute. All animal experiments were conducted with sex- and age-matched mice, and animal studies were compliant with all applicable provisions established by the Animal Welfare Act and the Public Health Services Policy on the Humane Care and Use of Laboratory Animals.

### *A. fumigatus* Strains and Culture

The strain used in these studies was a keratitis clinical isolate obtained from the Bascom-Palmer Eye Institute, *A. fumigatus* BP, whereas the strain used for corneal infection experiments was the Af293-expressing red fluorescent protein used in our prior studies (12). Fungi were cultured on Sabouraud dextrose agar at 37°C for 3–5 days for sporulation, and conidia were isolated by disruption in PBS and filtration through sterile cotton gauze. For hyphal killing experiments, conidia were grown in Sabouraud dextrose broth (SDB), at 3,000 conidia/well of 96-well plate until germination (6 h), washed in sterile PBS and co-incubated with neutrophils. For AspHE preparation, conidia were grown in 500 ml SDB at 37°C for 5 days. SDB was removed from the fungi by vacuum filtration, and fungal mass was homogenized in a mortar and pestle under liquid nitrogen. Fungal homogenate was resuspended in RPMI 1640 without phenol red and filtered through a 70 µM cell strainer. Protein content was measured by BCA assay (ThermoFisher), and AspHE stocks were stored at −20°C.

### Neutrophil Isolation

Human neutrophils were isolated from the blood of healthy donors age 18–50. All human studies were approved by the University of California, Irvine institutional review board. Whole blood was mixed 1:1 with 3% dextran-PBS for 20 min to sediment red blood cells. The upper layer was overlaid on Ficoll Hypaque (GE Healthcare) and centrifuged at 500 × *g* for 25 min. The pellet containing granulocytes and RBCs was treated with 1× RBC Lysis Buffer (eBioscience) and spun at 300 × *g* for 5 min to obtain a granulocyte pellet. Cells were resuspended in RPMI 1640 and counted. Typical purity was >95%. Total bone marrow was isolated from the tibias, and femurs of mice and neutrophils were purified using the EasySep mouse neutrophil enrichment kit (Stemcell), routinely yielding >90% neutrophil population.

### SYTOX Green Assay for Extracellular DNA

Neutrophils were resuspended in RPMI 1640, no phenol red (Life Technologies) plus 10% FBS. Murine GM-CSF (20 ng/ml, R&D Systems) and 200 µM CaCl_2_ were added to media for overnight mouse neutrophil experiments. In some assays, neutrophils were incubated at 37°C with inhibitors or blocking antibodies for 30 min before stimulation. Diphenyliodonium (DPI, Sigma-Aldrich) and a 10 mM stock solution in DMSO were prepared. The PAD4 inhibitors GSK484 and GSK106 described in Ref. (19) were purchased from Cayman chemical, and 40 mM of stock solutions was prepared in DMSO. Blocking antibody for CR3, LEAF purified antihuman/mouse CD11b, M1/70 was purchased from BioLegend. Anti-Dectin-1 22H8 blocking antibody was purchased from Invivogen. 2 × 10^5^ cells/well for human or 1 × 10^5^ cells/well for mice were added to black 96-well plates with optical bottoms (Corning) ± RPMI only, PMA (25 nM), particulate β-glucan (curdlan) (100 µg/ml) or AspHE (100 µg/ml) and 1 µM SYTOX Green nucleic acid stain (Life Technologies). Plates were incubated at 37°C, 5% CO_2_ in a Biotek Cytation 5 imaging plate reader, and fluorescence was measured every 30 min at 504/523 nm for up to 24 h. Plates were imaged at 40–200× using brightfield or GFP filters.

### Quantification of Reactive Oxygen

Neutrophils were resuspended in RPMI 1640 without phenol red (Life Technologies). In some assays neutrophils were incubated at 37°C with inhibitors or blocking antibodies for 30 min before stimulation. 2 × 10^5^ cells/well were added to black 96-well plates with optical bottoms (Corning) ± RPMI only or curdlan (100 µg/ml) plus 500 µM luminol (Sigma-Aldrich) in the absence of serum, and luminescence was measured every 2 min for 2 h on a Biotek Cytation 5.

### Immunocytochemistry and Fluorescence Imaging

Neutrophils were resuspended in RPMI 1640, no phenol red, 2% FBS at 2 × 10^6^ cells/ml. In some assays, neutrophils were incubated at 37°C with cytokines, inhibitors or blocking antibodies for 30 min before stimulation. Cells were plated on glass bottom 8-well chamber slides (Ibidi) or poly-l-lysine coated glass coverslips (Neuvitro) ± RPMI only or curdlan (100 µg/ml) for up to 24 h. Media were removed, and cells were fixed for 30 min in 4% formaldehyde in PBS. Cells were permeabilized in 0.1% Triton-X100 in PBS for 15 min and washed in PBS. Cells were stained for H3Cit (Abcam 5103) 1:100 and NE (SCBT) at 1:50 overnight at 4°C. Slides were washed in PBST, and donkey anti-rabbit Alexafluor 488 or donkey anti-goat Alexafluor 568 (Life Technologies) was added for 1 h at RT. Slides were washed in PBST, and 100 µl of PBS-DAPI was added to slides. Slides were imaged at 200× on a Biotek Cytation 5 using GFP, RFP and DAPI filters and on a Leica confocal at 600×.

### S100A8 ELISA

Mouse neutrophils were incubated in RPMI 1640 + 10% FBS + 20 ng/ml GM-CSF (R&D Systems) ± curdlan (100 µg/ml) or AspHE (100 µg/ml) at 1 × 10^5^ cells/well in 96-well plates as described (12). Plates were incubated at 37°C for 2 or 16 h, centrifuged at 300 × *g* for 3 min, and cell-free supernatants were collected. S100A8 was measured by ELISA (R&D Systems) according to the manufacturer’s directions.

### Fungal Growth Inhibition Assay

These assays were performed as described recently (12). Briefly, *A. fumigatus* conidia were grown to a hyphal stage in 96-well plates and incubated 18 h at 37°C with 2 × 10^5^ neutrophils/well. Following incubation, the supernatant was removed, and wells were stained with 50 µl Calcofluor white chitin stain (Sigma-Aldrich) for 10 min. Wells were washed three times in ddH_2_O, and fluorescence was read on a Biotek Cytation 5 at 360/440 nM.

### *A. fumigatus* Corneal Infection

Mice were anesthetized, the corneal epithelium was penetrated with a 30 G needle, and 2 µl containing 50,000 conidia (dsRed Af293) were injected into the corneal stroma using a 33 G Hamilton syringe as described (12).

After 48 h, mice were euthanized, and corneas were imaged by brightfield to detect corneal opacity, and by fluorescence to detect dsRed hyphae. Images were analyzed, and corneal opacity and dsRed were quantified using Metamorph with the parameters described by us in detail (11, 34). Whole eyes were homogenized using a Retsch Mixer Mill bead homogenizer (QIAGEN Sciences, Germantown, MD, USA), and colony forming units were counted manually.

### Flow Cytometry

Corneas were carefully dissected to remove adherent iris material and digested 1 h in collagenase type 1 (80u/cornea; Sigma-Aldrich, cat no 234153). Total cells were collected and incubated with Fc block anti-mouse CD16/CD32 (clone 93, 16-0161-86; eBioscience), followed by anti-mouse neutrophil antibody (Ly6G, NIMP-R14-PE, ab125259; Abcam, Cambridge, MA, USA), and anti-CD11b-APC FAB1124A (R&D Systems, Minneapolis, MN, USA). We also used CD45-FITC (30-F11), Ly6C APC-Cy7 (HK1.4), and Ep-CAM-PECy7 (G8.8), all from BioLegend. Total cells were examined in an ACEA Novocyte™ flow cytometer and analyzed following appropriate compensation.

### Immunofluorescence Staining of Mouse Corneas

Whole corneas were excised and immediately embedded in optimal cutting temperature (Thermo Fisher Scientific). Frozen sections (10 µm) were mounted on Superfrost™ Plus™ slides and stored at −80°C. Before staining, the slices were placed on a 37°C hot plate for 10 min followed by submersion in acetone for 10 min at −20°C. The slides were washed in PBS twice, permeabilized 5 min in 0.1% Triton-X/PBS, and incubated at room temperature for 1 h in blocking buffer containing 2% BSA and 0.1% Tween 20 with 1:200 Fc block (Anti-CD16/32; Tonbo Biosciences), and 10% donkey serum (Vector Labs).

Corneal sections (5 µm) were then stained at room temperature for 2 h with Ly6G antibody NIMPR14 at 40 µg/ml (Abcam) and 1:50 Rabbit polyclonal antibody to histone H3 (citrulline R2 + R8 + R17) (Abcam). Slides were rinsed three times in PBS, and then secondary antibodies, donkey anti-rabbit Alexa Fluor 555 or goat anti-rat Alexa Fluor 488, were applied at 1:1,000 (Invitrogen) for 1 h at room temperature. Slides were rinsed and mounted in Fluoromount with DAPI (Thermo Fisher Scientific). All images were acquired within 24 h.

### Statistical Analysis

Experimental data from *in vitro* and *in vivo* studies were analyzed for significance from at least three independent experiments. Statistical significance for multiple parameters was determined using one-way ANOVA with Tukey *post hoc* analyses, or using Student’s *t*-test when only two parameters were compared.

All statistical analyses were performed with GraphPad Prism software, v6.0c (La Jolla, CA, USA). A *p* value < 0.05 was considered significant as indicated in the figure legends (*p ≤ 0.05, **p ≤ 0.01, and ***p ≤ 0.001).

## Ethics Statement

All animals were used in accordance with the guidelines of the Case Western Reserve University and University of California, Irvine Institutional Animal Care and Use Committee (IACUC). Whole blood was collected from healthy donors between ages of 18 and 65 years in accordance with the Declaration of Helsinki guidelines and the Institutional Review Board of the University of California (Irvine, CA, USA).

## Author Contributions

HC, SA, MM, YS, and EG: experimental design, performance, and data analysis. HC and EP: manuscript preparation.

## Conflict of Interest Statement

None of the submitted work was carried out in the presence of any personal, professional, or financial relationships that could potentially be construed as a conflict of interest.
