# Supplementary figures and images for: Protein Deiminase 4 and CR3 Regulate Aspergillus fumigatus and β-Glucan-Induced Neutrophil Extracellular Trap Formation, but Hyphal Killing Is Dependent Only on CR3
Source: Front Immunol. 2018 May 29;9:1182. doi: 10.3389/fimmu.2018.01182 (PMC5986955; doi:10.3389/fimmu.2018.01182)

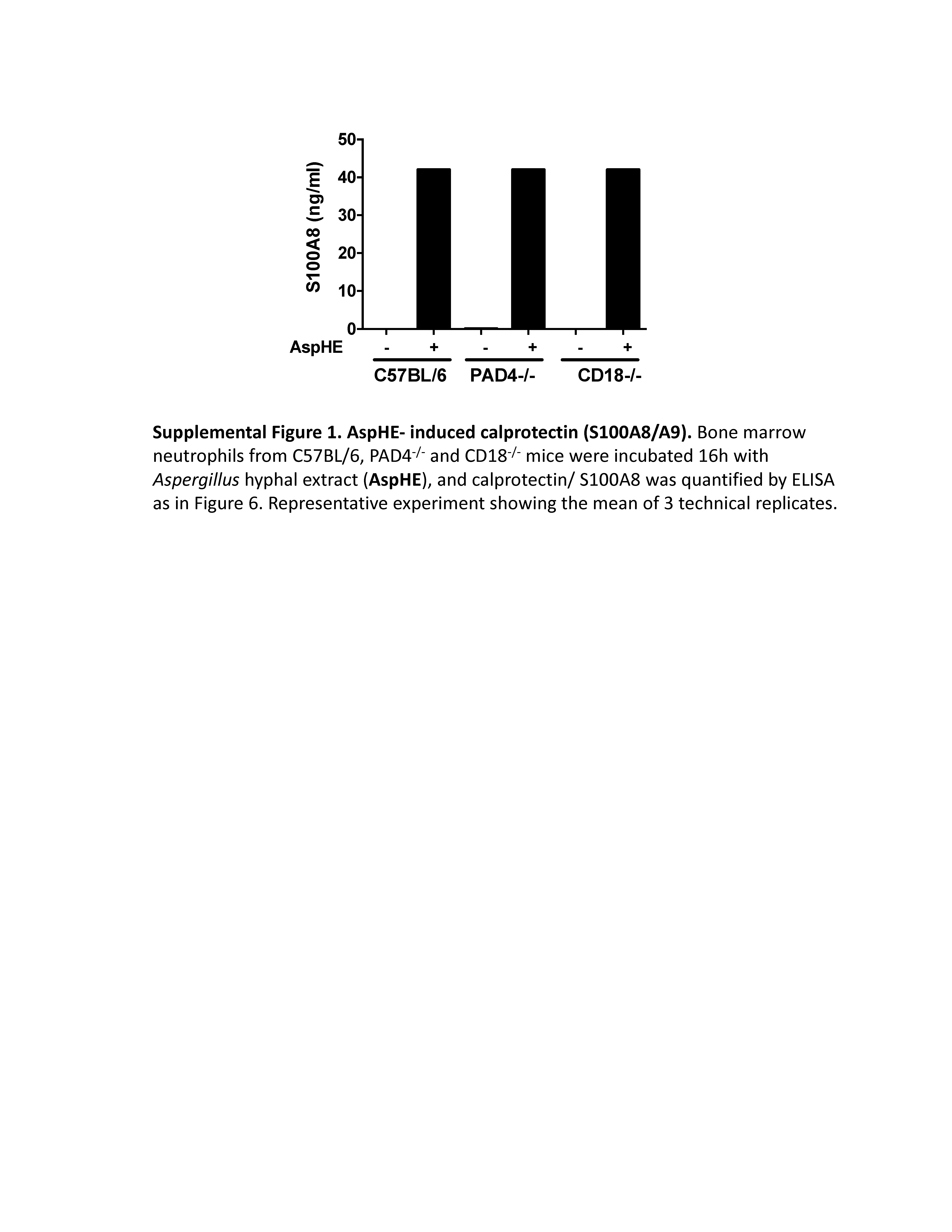

Supplement: Supplementary file 1 [file image_1.tiff]

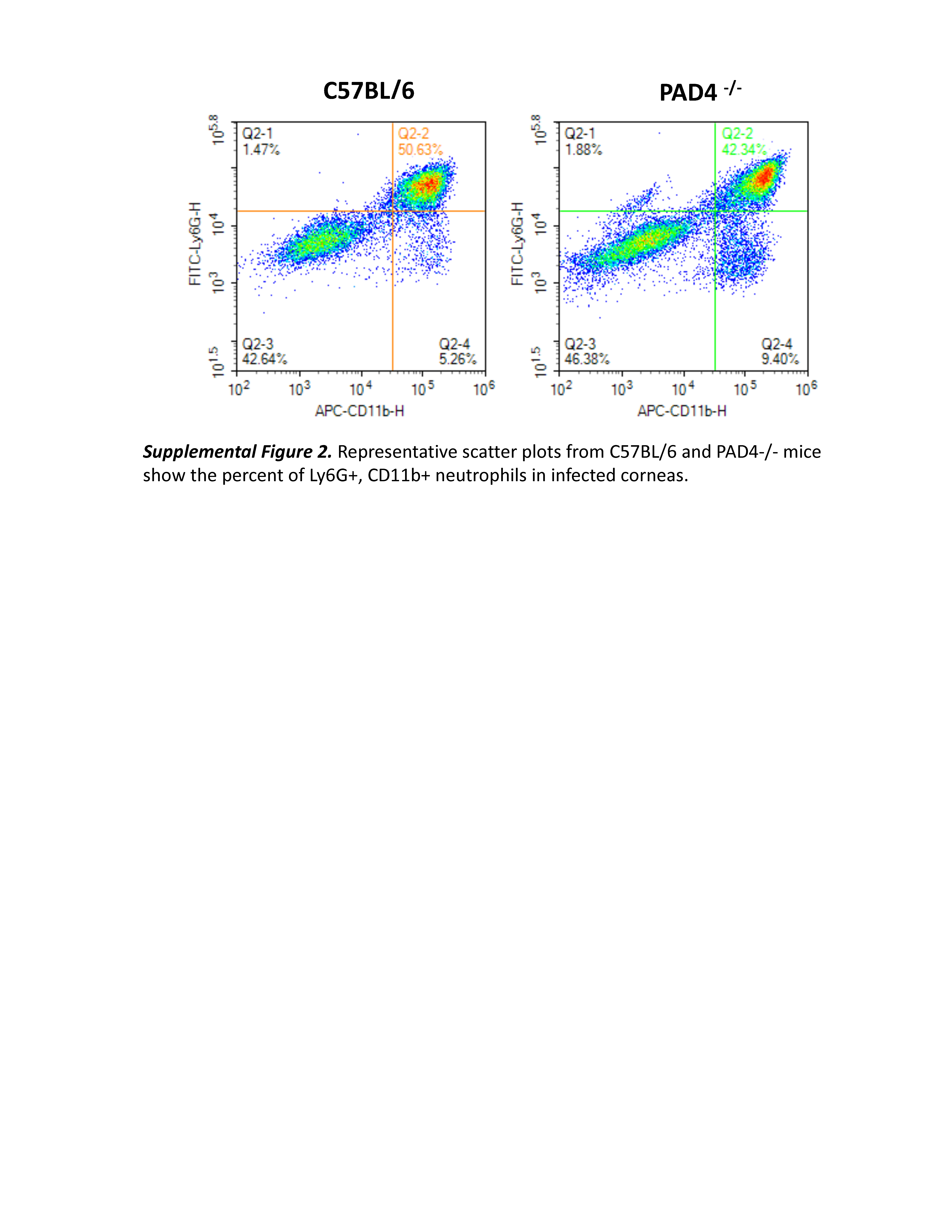

Supplement: Supplementary file 2 [file image_2.tiff]

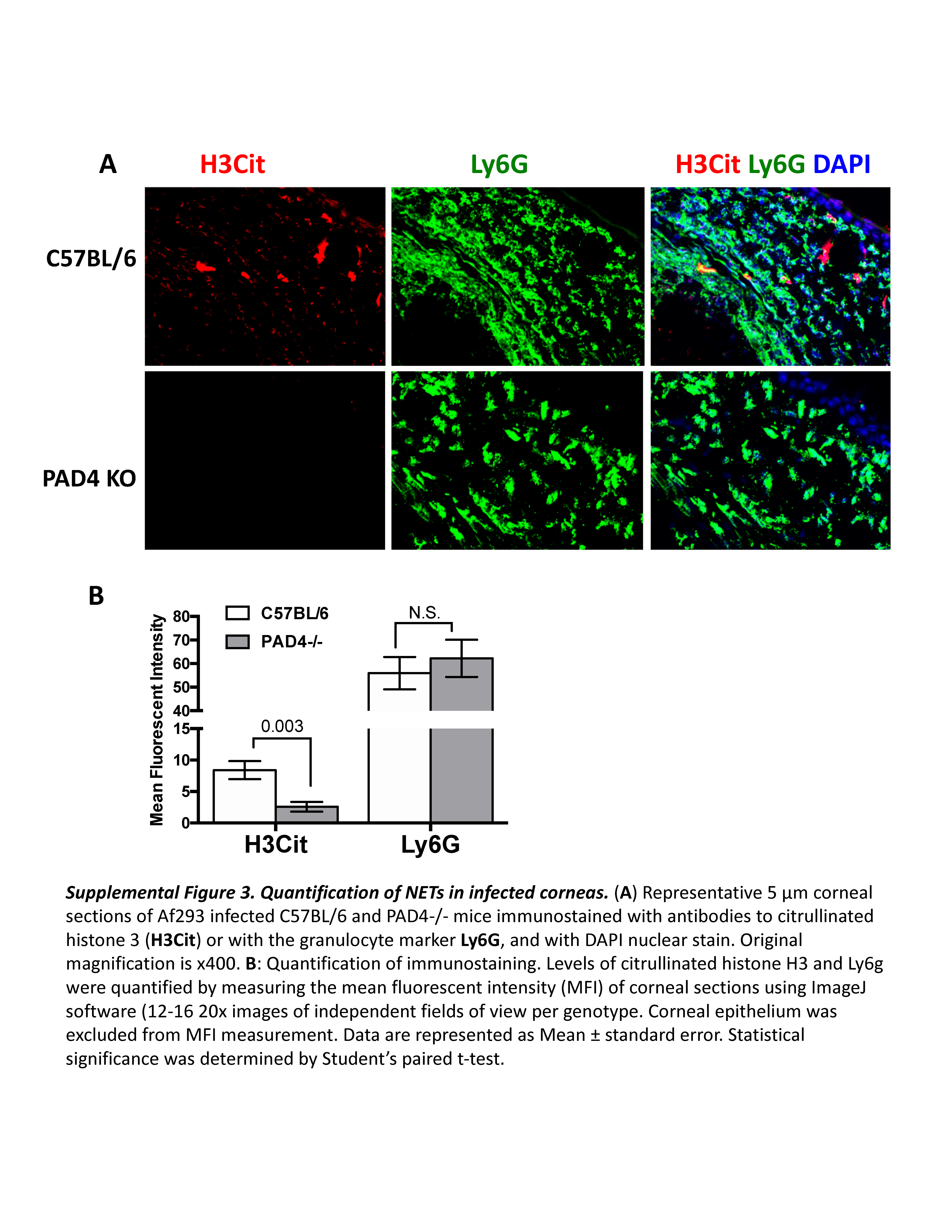

Supplement: Supplementary file 3 [file image_3.tiff]
